# Assessment of fine-scale parameterizations at low latitudes of the North Pacific

**DOI:** 10.1038/s41598-018-28554-z

**Published:** 2018-07-06

**Authors:** Chang-Rong Liang, Xiao-Dong Shang, Yong-Feng Qi, Gui-Ying Chen, Ling-Hui Yu

**Affiliations:** State Key Laboratory of Tropical Oceanography, South China Sea Institute of Oceanology, Chinese Academy of Sciences, Guangzhou, 510301 China

## Abstract

Fine-scale parameterizations based on shear and stratification are widely used to study the intensity and spatial distribution of turbulent diapycnal mixing in the ocean. Two well-known fine-scale parameterizations, Gregg–Henyey–Polzin (GHP) parameterization and MacKinnon–Gregg (MG) parameterization, are assessed with the full-depth microstructure data obtained in the North Pacific. The GHP parameterization commonly used in the open ocean succeeds in reproducing the dissipation rates over smooth topography but fails to predict the turbulence over rough topography. Failure of GHP parameterization over rough topography is attributed to the deviation of internal wave spectrum from the Garrett–Munk (GM) spectrum. The internal wave field over rough topography is characterized by energetic intermediate-scale and small-scale internal waves that are not described well by the GM model. The MG parameterization that is widely used in coastal environments is found to be successful in reproducing the dissipation rates over both smooth and rough topographies. The efficacy of GHP and MG parameterizations in evaluating the dissipation rates has been assessed. The result indicates that MG parameterization predicts the magnitude and variability of the dissipation rates better than the GHP parameterization.

## Introduction

An outstanding question in oceanography is the intensity and spatial distribution of turbulent diapycnal mixing in the ocean. Turbulent diapycnal mixing modifies water masses, maintains ocean stratification and drives the meridional overturning circulation^1–4^. With one-dimensional advection-diffusion model, *Munk* [1966] argued that an average diapycnal diffusivity (*κ*) of *O* (10^−4^ m^2^ s^−1^) is required to maintain the observed abyssal stratification and drive the meridional overturning circulation. But an assessment of whether the globally averaged diapycnal diffusivity can reach 10^−4^ m^2^ s^−1^ at any depth is prevented by the difficulty of direct measurements of diapycnal mixing, with vast regions of the ocean remaining essentially unsampled. Thus researchers typically rely on fine-scale parameterization; a technique allows us to estimate diapycnal diffusivity from observed temperature, salinity, and current velocity. The most commonly used fine-scale parameterization in the open ocean is the Gregg–Henyey–Polzin (GHP) parameterization^5–7^. Studies relied on this parameterization^8–14^ have shown that enhanced turbulent diapycnal mixing (*κ* ≥ 10^−4^ m^2^ s^−1^) occurs over rough topography. For example, using GHP parameterization, *Sloyan* [2005] found that turbulent diapycnal mixing in the southern hemisphere oceans is vertically and spatially non-uniform. Enhanced diffusivities (*κ* ≥ 10^−4^ m^2^ s^−1^) are found over rough topography. With the same parameterization, *Wu et al*.^14^ reported that the spatial distribution of turbulent diapycnal mixing in the Southern Ocean is controlled by the topography, by means of its interaction with the Antarctic Circumpolar Current. *Jing et al*.^10^ studied the turbulent diapycnal mixing in the subtropical northwestern Pacific with the GHP parameterization. Enhanced diffusivities of *O* (10^−4^ m^2^ s^−1^) are found over rough topography in which topography interactions play an important role. These studies based on GHP parameterization have greatly aided our knowledge of the intensity and distribution of turbulent diapycnal mixing in the open ocean. However, in the development of GHP parameterization some assumptions have been made that may not be entirely appropriate for the oceanic internal wave field. GHP parameterization was developed based on the assumption that interactions between internal waves act to steadily transport energy from large scales to small scales at which waves break due to shear or convective instabilities^5,6,8^. Its predication is typically evaluated for the internal wave field as described by the Garrett-Munk (GM) model^6,15^. An assessment of fine-scale parameterizations points out that GHP parameterization of turbulent dissipation rates in the deep ocean become erroneous near topographic features where internal wave spectra deviate from GM spectra^16^. Thus using GHP parameterization near topographic features should be cautious and an appropriate fine-scale parameterization for regions where internal wave spectra deviate from GM spectra is needed to better understand the intensity and spatial distribution of turbulent diapycnal mixing.

Recently, *MacKinnon and Gregg* [2003a] proposed an analytical model, known as the MacKinnon-Gregg (MG) parameterization, by incorporating the internal wave properties over the continental shelf. It is found to be appropriate in coastal environments where the internal wave field might not be described well by the GM model due to the topography interactions, wind stress, and internal solitary waves^17–23^. For example, *MacKinnon and Gregg* [2003a; 2005b] verified that MG parameterization succeeds in predicting the dissipation on the New England continental shelf where the wave evolution is controlled by wind stress and bottom drag^24,25^. *Van der Lee and Umlauf* [2011] reported that MG parameterization is applicable in the Bornholm Basin of the southern Baltic Sea where the internal wave field is governed by low-mode near-inertial wave motions. *Xie et al*.^23^ found that MG parameterization can be applied in the central Bay of Biscay where the internal wave field is characterized by large-amplitude internal tides and internal solitary waves. *Shang et al*.^22^ found that MG parameterization can be applied in the upper ocean of the South China Sea.

Though the MG parameterization was proved to be applicable in coastal environments, few studies have been made to assess this parameterization in the open ocean. Internal wave fields over rough topography in the open ocean are strongly influenced by the topography interactions. Features of these internal wave fields are likely to be consistent with those in coastal environments. Thus it is possible that MG parameterization can overcome the defects of GHP parameterization and work well over rough topography in the open ocean. To confirm this possibility, we assess the GHP and MG parameterizations with the full-depth microstructure data obtained in the North Pacific. The assessment would provide a useful reference for researchers on choosing fine-scale parameterizations to explore the intensity and spatial distribution of turbulent diapycnal mixing in the open ocean.

## Results

**Figure 1:**
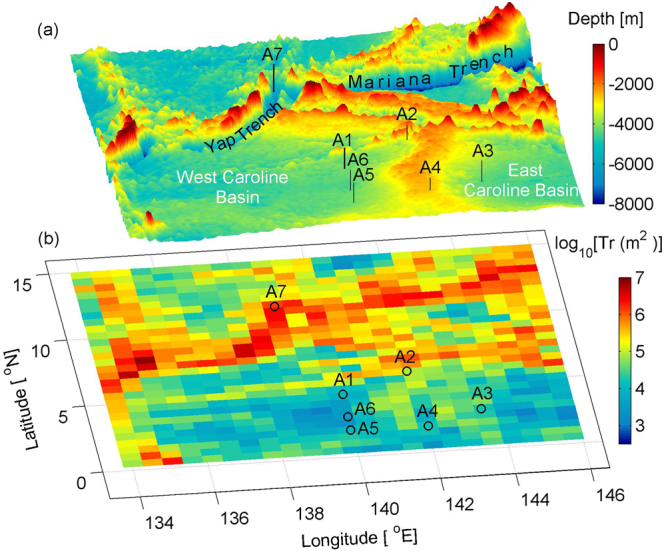
(**a**) Three-dimensional distribution of bottom topography. (**b**) Topographic roughness with the stations shown.

Our Field observations were conducted at seven stations (A1–A7) in the North Pacific on cruises from October to November in 2015 (Fig. 1a). The study region hosts a wide range of topographic features including featureless abyssal basins, flat ridges, and complex trenches. Detail information about the stations, such as observation time, latitude/longitude, water depths, and topographic roughness (*Tr*), is given in Table 1. *Tr* represents the variance of bottom height^11^. The ETOPO2v2 (2-Minute Gridded Global Relief Data) were used to evaluate *Tr* in a 1/2° × 1/2° box which is approximately a 55 km × 55  km domain. Here the smooth topography is defined as regions with *Tr* less than 10^5^ m^2^, and the rough topography as that with value larger than 10^5^ m^2^. Stations A1–A6 were located over smooth topography where values of *Tr* range from 10^3^ to 10^5^ m^2^, and station A7 was located over rough topography where the value of *Tr* is 1.96 × 10^6^ m^2^ (Fig. 1b).

**Table 1:** Information of the stations in this study.

Station	Observation time	Latitude (°N)	Longitude (^o^E)	Depth (m)	Roughness (m^2^)
A1	2015-10-21 15:04	4.71	140.04	4283	9.54e + 03
A2	2015-10-23 02:05	6.13	141.88	2975	7.94e + 04
A3	2015-10-26 09:11	3.00	143.55	4284	2.51e + 04
A4	2015-10-29 02:30	1.97	142.05	2577	2.04e + 04
A5	2015-11-03 11:38	2.00	139.98	4176	9.27e + 03
A6	2015-11-04 05:28	3.00	140.00	4223	1.58e + 04
A7	2015-11-07 15:47	11.55	138.87	5849	1.96e + 06

**Figure 2:**
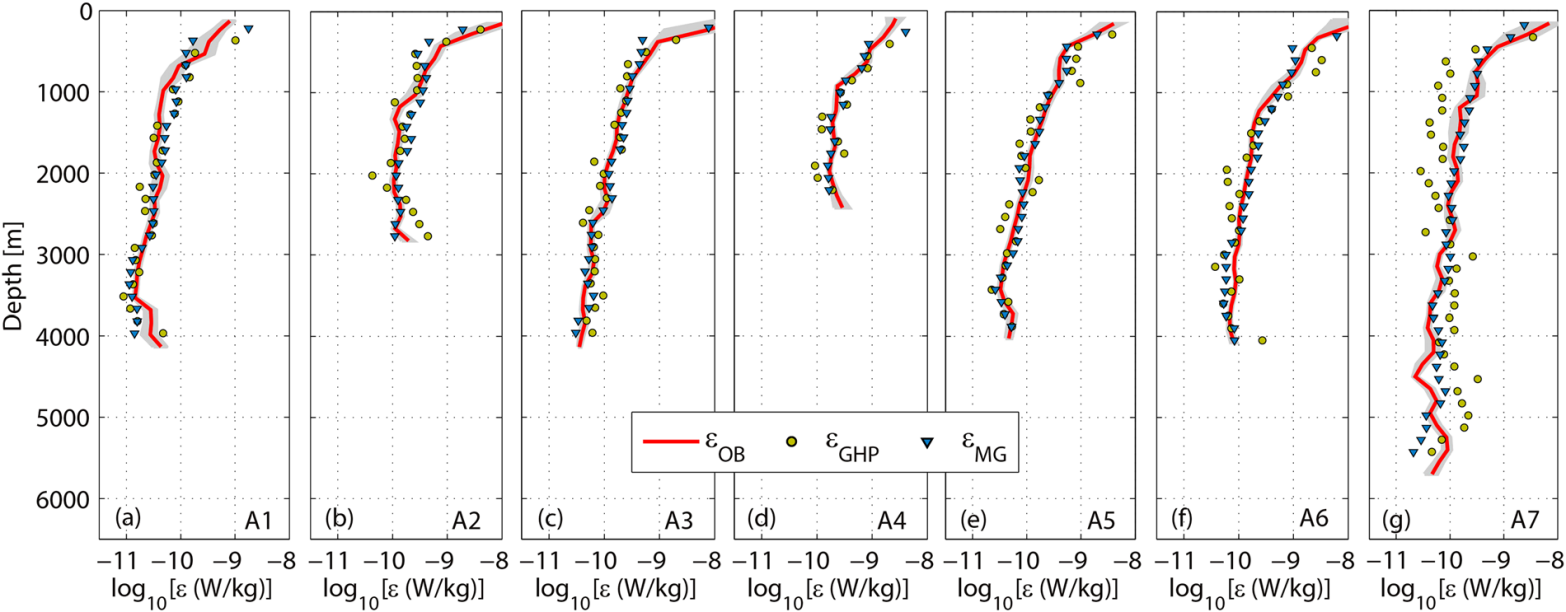
Profiles of dissipation rate from observation (*ε*_*OB*_), MG parameterization (*ε*_*MG*_), and GHP parameterization (*ε*_*GHP*_). 95% bootstrapped confidence intervals on *ε*_*OB*_ are shaded in light gray.

Profiles of dissipation rate estimated from microstructure data (*ε*_*OB*_), GHP parameterization (*ε*_*GHP*_), and MG parameterization (*ε*_*MG*_) are shown in Fig. 2. To be consistent with the resolution of *ε*_*GHP*_, *ε*_*OB*_ and *ε*_*MG*_ profiles have been broken into half-overlapping 300-m-long segments (starting from the bottom) and averaged onto the 150-m grid. *ε*_*OB*_ decease with increasing depth from *O* (10^−9^ W kg^−1^) in the upper layer to *O* (10^−11^ W kg^−1^) in the deep layer. Both GHP and MG parameterizations succeed in predicting the decreasing trend of the dissipation rates at stations A1–A6 (Fig. 2a–f). However, profiles of *ε*_*GHP*_ and *ε*_*MG*_ from station A7 display different patterns (Fig. 2g). *ε*_*MG*_ show a decreasing trend with increasing depth as *ε*_*OB*_ while *ε*_*GHP*_ trend to slightly increase with increasing depth below 500 m where *ε*_*GHP*_ deviate from the observed values.

**Figure 3:**
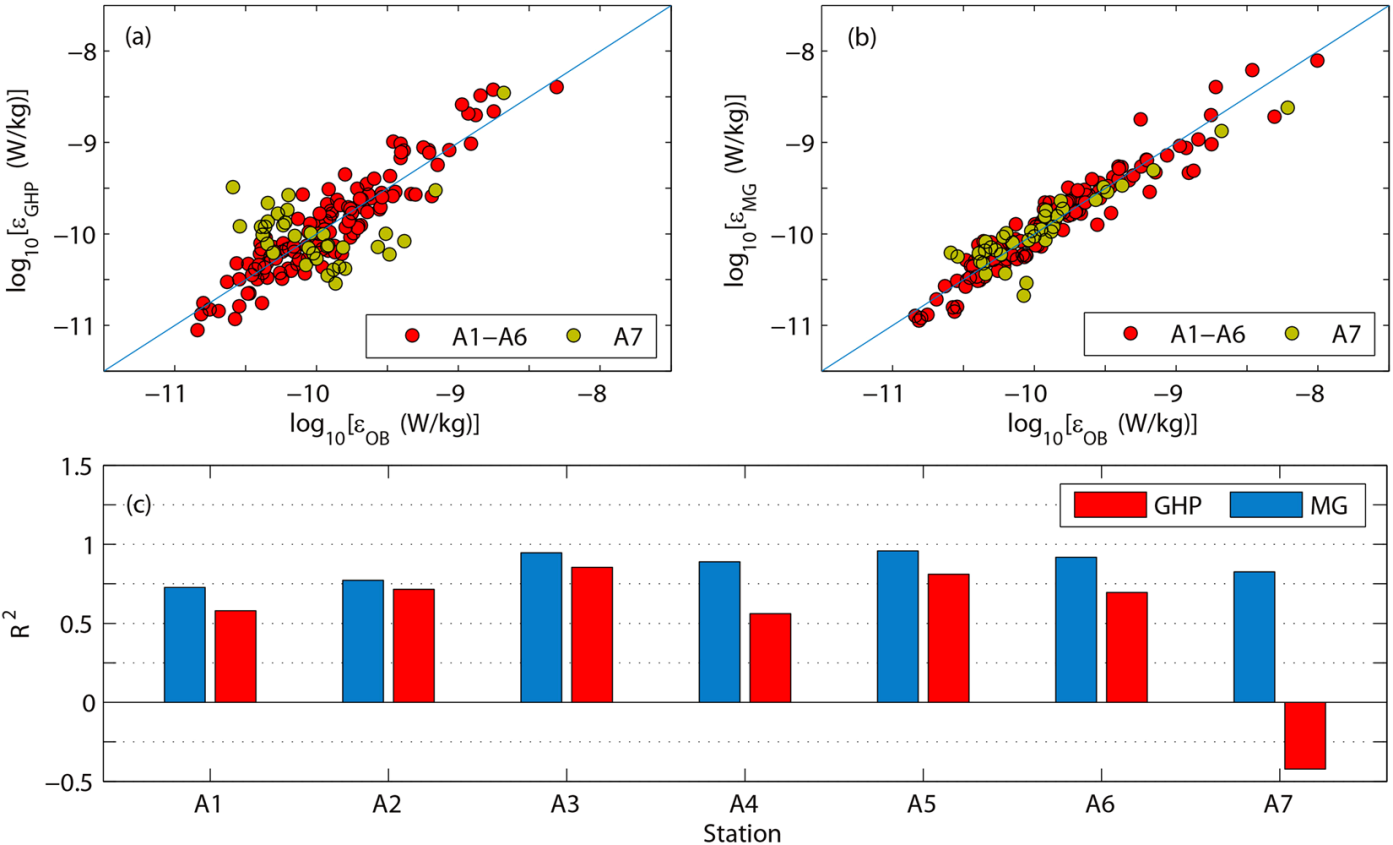
Observed dissipation plotted against (**a**) GHP and (**b**) MG dissipation. The red circles are the data from stations A1–A6. The yellow circles are the data from station A7. Straight lines indicate the one-to-one relations. (**c**) Coefficients of determination *R*^2^ for the observed stations A1–A7.

To evaluate the success of GHP and MG parameterizations in predicting the magnitude of the dissipation rates, we consider relation between parameterized and observed dissipation rates (Fig. 3a,b). If parameterizations succeed in predicting the magnitude of the dissipation rates, the relation between parameterized and observed dissipation rates will be close to the one-to-one relations of log_10_(*ε*_*OB*_) = log_10_(*ε*_*GHP*_) and log_10_(*ε*_*OB*_) = log_10_(*ε*_*MG*_). *ε*_*GHP*_ from stations A1–A6 (Fig. 3a) show a good relation to *ε*_*OB*_, like the one-to-one relation. However, *ε*_*GHP*_ from station A7 show a bad relation to *ε*_*OB*_ with most of *ε*_*GHP*_ deviating largely from the one-to-one relation. These observations suggest that GHP parameterization predicts the magnitude of the dissipation rates at stations A1–A6 better than that at station A7. For MG parameterization (Fig. 3b), *ε*_*MG*_ from all of the stations (A1–A7) show a good relation to *ε*_*OB*_ with all of the values gathering around the one-to-one relation. Comparing Fig. 3a,b, one can see that MG parameterization predicts the magnitude of the dissipation rates better than the GHP parameterization.

To assess the efficacy of GHP and MG parameterizations in reproducing the variability of the dissipation rates, we calculate the coefficient of determination (*R*^2^). *R*^2^ is the ratio of the difference between the variance of the observed values and the variance of the residuals from the parameterization to the variance of the observed values^26^. It can be interpreted as the proportion of the variability of the observed values that can be explained by the parameterized values. *R*^2^ for each station are shown in Fig. 3c. At stations A1–A6, values of ${R}_{MG}^{2}$ and ${R}_{GHP}^{2}$ are positive and larger than 0.5, which indicates that both GHP and MG parameterizations can effectively predict the variability of the dissipation rates at stations A1–A6. At station A7, ${R}_{MG}^{2}$ is still positive and larger than 0.5 while ${R}_{GHP}^{2}$ is negative. A negative ${R}_{GHP}^{2}$ suggests that GHP parameterization fails to predict the variability of dissipation rates at station A7. For all of the stations, the values of ${R}_{MG}^{2}$ are larger than that of ${R}_{GHP}^{2}$, which implies that MG parameterization predicts the variability of the dissipation rates better than the GHP parameterization.

The above analysis indicates that MG parameterization succeeds in predicting the dissipations at all of the stations while GHP parameterization only succeeds at stations A1–A6. The success of fine-scale parameterizations greatly depends on the internal wave fields. To explore the feature of the internal wave field at each station, baroclinic velocity was formally decomposed onto a set of orthogonal vertical modes. The vertical structure of each mode is governed by^24,27^

1 $$\begin{array}{c}{{\rm{\Psi }}^{\prime\prime} }_{j}(z)=-\,\frac{{N}^{2}(z)}{{c}_{j}^{2}}{{\rm{\Psi }}}_{j}(z),\\ {{\rm{\Psi }}}_{j}(-H)={{\rm{\Psi }}}_{j}(0)=0,\end{array}$$

where *c*_*j*_ is the separation constant (eigenvalue), and *H* is the water depth. The vertical mode shapes are calculated from numerical solution of equation (1) using stratification profile. Vertical velocity and vertical displacement associated with each mode are proportional to Ψ_*j*_, while the horizontal velocity is proportional to dΨ_*j*_/dz. Baroclinic velocity data were fit to the baroclinic modes using a least square regression.

**Figure 4:**
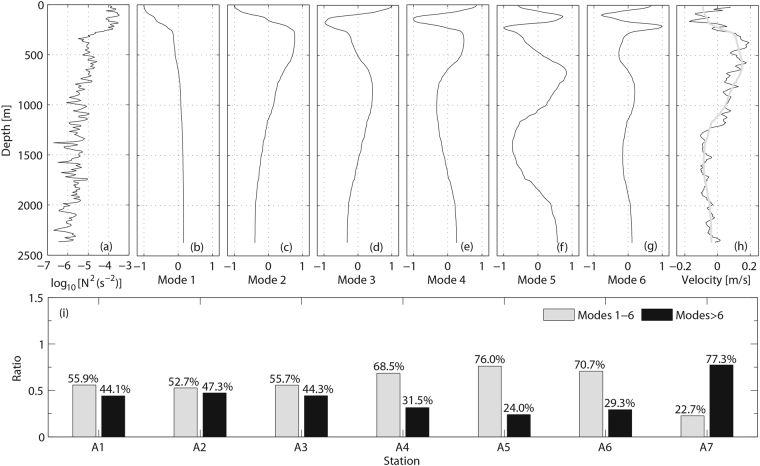
(**a**) Stratification (*N*^*2*^) profile of station A4. (**b**–**g**) Shapes of first six baroclinic modes based on a numerical solution of equation (1). (**h**) Measured northward velocity (black) and sum of the first six baroclinic mode fits (gray) for the velocity. (**i**) Ratios of the energy in low modes (modes 1–6, gray) and high modes (modes > 6, black) to the total energy.

**Figure 5:**
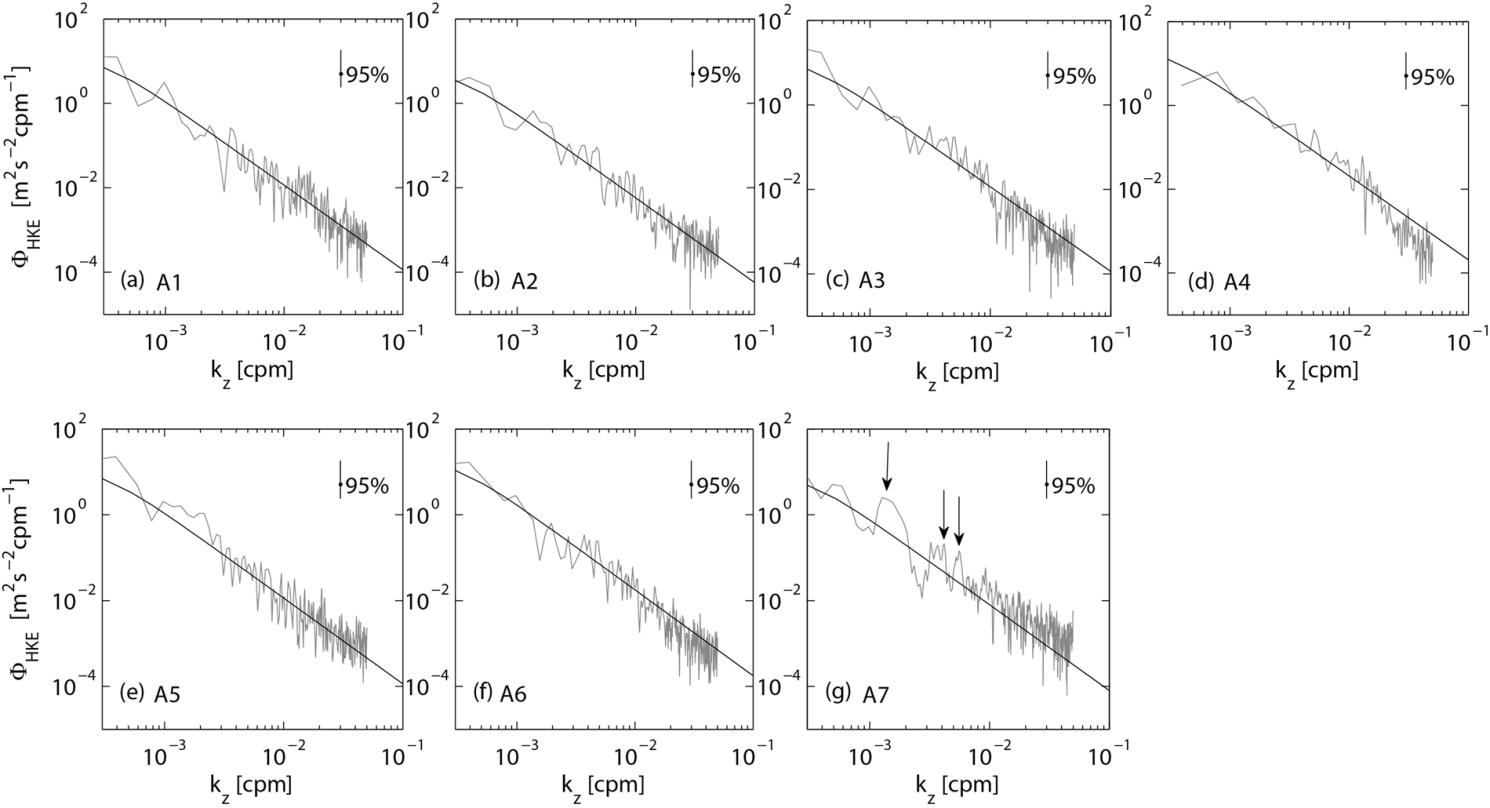
Observed (gray) and GM (black) dropped spectra. The vertical lines in the upper right hand corner represent the 95% statistical significance level. The arrows in (g) indicate the spectrum peaks.

Figure 4a shows a sample stratification (*N*^2^) profile from station A4. Stratification is strong in the upper 300 m (*N*^2^ ~ 10^−4^ s^−2^) and become weak below (*N*^*2*^ ~ 10^−6^–10^−5^ s^−2^). Figure 4b–g show the first six baroclinic modes calculated with the stratification profile in Fig. 4a. The modes have been normalized. The velocity fitted from the first six modes is shown in Fig. 4h. The modal fit captures the dominant low-mode signal well, though it does not reproduce the small-scale fluctuations. To look at the distribution of horizontal kinetic energy in modes, we calculate the ratio (*Ra*) of the energy in low modes (modes 1–6) to the total energy. The ratio of the energy in high modes (modes >6) to the total energy is given as 1-*Ra*. The result is shown in Fig. 4i. Note that background flows associated with mean current or mesoscale eddies may also make important contribution to the observed horizontal velocity. Here we remove the background flows by removing the depth-mean of each velocity component. This method works well though it might not remove the background flows completely. As one can see from Fig. 5 that large-scale (>1000 m) motions are roughly consistent with the GM spectrum. The internal wave fields are dominated by low-mode internal waves at stations A1–A6 with the energy in the first six modes accounting for more than half of the total energy. The Ratios even exceed 70% at stations A5 and A6. However, a different energy distribution is found at station A7. Low-mode internal waves are no longer the dominant components. Only 22.7% energy are captured by the first six modes, and 77.3% energy are from high modes. These observations indicate that high-mode internal waves are more active at station A7 than other stations.

The above modal analysis indicates that internal wave fields at different stations show different features. The internal wave field at station A7 is characterized by energetic high-mode internal waves. To find out whether the internal wave fields can be described well by the GM model, we compare the observed spectra with the GM spectra. GM model assumes that the frequency and vertical mode distributions of wave energy are separable functions^28^. Dropped spectrum of horizontal velocity is given as

2 $$\begin{array}{c}D{S}_{GM}=\frac{3}{4}E{b}^{2}{N}_{0}^{2}H(j),\\ H(j)=\frac{1}{({j}^{2}+{j}_{\ast }^{2})}\sum _{1}^{{\rm{\infty }}}\frac{1}{{j}^{2}+{j}_{\ast }^{2}},\end{array}$$

where *E* is the non-dimensional energy parameter; *b* is the vertical length scale; *N*_0_ is the buoyancy frequency scale; and *j* is the mode number. The vertical wavenumber is given as ${k}_{z}=j\pi N/(b{N}_{0})$. The observed and GM dropped spectra are shown in Fig. 5. The observed dropped spectra at stations A1–A6 (Fig. 5a–f) roll off smoothly as ${k}_{z}^{-2}$ as predicted by GM model (equation 2). However, observation at station A7 (Fig. 5g) did not show a smooth decrease with vertical wavenumber as predicted by GM model. Instead, it shows some significant elevated peaks at wavenumber band of 10^−3^–10^−2^ cpm (indicated by the arrows). These elevated peaks correspond to the energetic intermediate-scale internal waves which cannot be described well by the GM model. In addition, the energy level at higher wavenumbers (*k*_*z*_ > 10^−2^ cpm, small-scale internal waves) is higher than the GM dropped spectrum, which indicates that more energy is transferred from large scales to small scales than that predicted by the GM model.

## Discussion

With the full-depth microstructure data obtained in the North Pacific, we have assessed the GHP and MG parameterizations. GHP parameterization succeeds in predicting the dissipation over smooth topography (stations A1–A6) but fails over rough topography (station A7). One possible explanation for the failure of GHP parameterization over rough topography is that the internal wave spectra over rough topography diverge from the GM model. GHP parameterization was developed based on the assumption that the waves are statistically stationary with respect to wave-wave interactions by which energy is transferred from large to small scales^7^. It is typically evaluated for the internal wave field with GM spectral shape, but inappropriate for the regions where the internal wave field deviates from the GM model^6,15,16^. Station A7 is characterized by energetic intermediate-scale and small-scale internal waves that are not described well by the GM model (Fig. 5). These energetic internal waves might be caused by topography interactions rather than the wave-wave interactions as predicted by GM model. Studies^29–32^ have indicated that topography interactions could push energy directly into high wavenumbers and account for the departure of the observed spectrum from the wave-wave interactions underlying the GM model. Station A7 is located at the intersection of the Yap Trench and Mariana Trench (Fig. 1) where the topography is rather rough (*Tr* = 1.96 × 10^6^ m^2^). Energetic high-mode internal waves (Fig. 4i), elevated peaks at wavenumber band of 10^−3^–10^−2^ cpm, and high energy level at large wavenumbers (>10^−2^ cpm) in the spectrum of station A7 (Fig. 5g) might result from topography interactions.

In the classical lee-wave problem^33^, a constant flow (e.g., geostrophic flow) in a stratified fluid over a variable bottom topography generates upward radiating internal waves. The vertical wave number of the generated waves is related to the horizontal wave number (*k*_*h*_) of topography^32^,

3 $${k}_{z}={k}_{h}\sqrt{\frac{{N}^{2}-{U}_{0}^{2}{k}_{h}^{2}}{{U}_{0}^{2}{k}_{h}^{2}-{f}^{2}}},$$

where *U*_0_ is a constant flow, *f* is the Coriolis frequency. *f* = 4.65 × 10^−6^ s^−1^ at station A7 and the observed area is characterized by topography with *k*_*h*_ of 10^−4^–10^−3^ cpm. Assuming *U*_0_ = 0.05 m s^−1^ and N = 10^−3^ s^−1^ (based on our observation), *k*_*z*_ is *O*(10^−2^ cpm). These internal waves might contribute to the high energy level at large wavenumbers (>10^−2^ cpm) in the spectrum of station A7 (Fig. 5g). In addition to constant flow, strong tidal currents (e.g., barotropic diurnal and semidiurnal tides) over topographic features with widths less than a tidal excursion can also cause internal waves, which can propagate away from the topography as the forcing frequency or high-frequency (superharmonics) internal waves^31,34,35^. The dispersion relationship for internal waves is

4 $${k}_{z}={k}_{h}\sqrt{\frac{{N}^{2}-{\omega }^{2}}{{\omega }^{2}-{f}^{2}}},$$

where *ω* is the frequency of internal waves. The observed area is characterized by strong diurnal and semidiurnal tidal currents^36,37^. Thus for generated internal waves with diurnal (*ω* ≈ 1.16 × 10^−5^ s^−1^) and semidiurnal (*ω* ≈ 2.31 × 10^−5^ s^−1^) frequencies, *k*_*z*_ is 10^−3^–10^−2^ cpm, which is consistent with the observed internal waves at wavenumber band of 10^−3^–10^−2^ cpm in the spectrum of station A7 (Fig. 5g).

**Figure 6:**
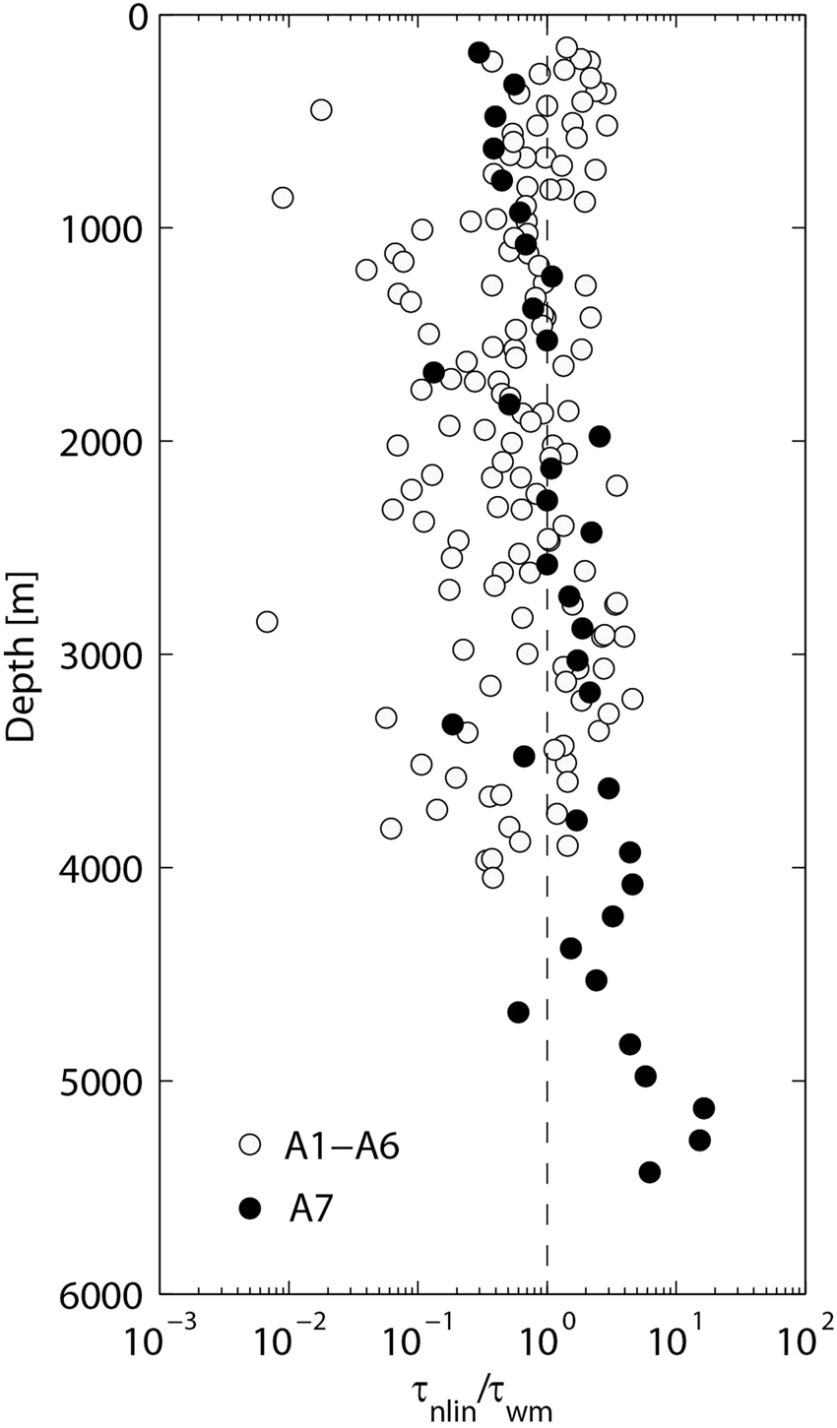
Ratio of time scales characterizing wave-wave and wave-mean interactions for all of the stations. The gray circles represent the ratio of stations A1–A6 and the black circles represent the ratio of station A7. The vertical dashed line represents the ratio equal to one.

Other possible reasons for the failure of GHP parameterization over rough topography are wave-mean interactions. If the wave-mean interactions dominate nonlinear transports, significant biases are possible^38^. In order to gauge the extent to which wave-mean interactions may be significant, it is convenient to evaluate the ratio of time scales characterizing wave-wave and wave-mean interactions. Invoking the ray tracing equations and assuming waves are randomly aligned with the mean shear, the ratio can be shown to be^38^

5 $$\frac{{\tau }_{nlin}}{{\tau }_{wm}}=8\frac{\bar{S}}{N}\frac{{k}_{z}^{c}}{{k}_{z}},$$

where $\bar{S}$ represents the mean shear, ${k}_{z}^{c}$ represents the cutoff wave number. Equation (5) states that, if the waves are randomly aligned with the mean shear, wave-mean interactions dominate the spectral energy transport in vertical wave number space at ${k}_{z}^{c}$ for mean shears in excess of *N*/8. Figure 6 shows the ratio for vertical wave numbers equal to ${k}_{z}^{c}$. Although many of the values from stations A1–A6 are greater than one, GHP parameterization is effective at these stations. It might be understood that the finescale wavefield tends to be aligned normal to the mean shears. These phenomena also occurred in other observations^6,39^, in which the ratio exceeded one and finescale parameterizations were still applicable. Here, it is necessary to compare the ratio between stations A1–A6 and station A7. Ratio of station A7 is *O*(1) at most of depth and there is no significant discrepancy among the stations, which suggests that wave-mean interactions are not the major cause of the failure of GHP parameterization at station A7.

Although GHP parameterization fails over rough topography where the internal wave field deviates from the GM model, our observation indicates that the MG parameterization succeeds in reproducing the dissipation not only over smooth topography but also over rough topography. In addition, our statistical results indicated that the MG parameterization predicts the magnitude and variability of the dissipation rates much better than the GHP parameterization. MG parameterization was developed based on the assumption that there is no statistical relationship between shear in low- and high-mode waves, and the strength of low-mode shear is decoupled from properties of high-mode waves^18,19^. MG parameterization was first proposed to predict the dissipation rates over the New England shelf^18^. Later it is found to be suitable for the coastal environments^19–21^ where the internal wave fields are more complex than that predicted by the GM model. Here we further verify that MG parameterization is also applicable in the deep ocean where the internal wave field deviates from the GM model.

GHP parameterization was most commonly used to explore the intensity and spatial distribution of turbulent diapycnal mixing in the open ocean. With the GHP parameterization, elevated turbulent diapycnal mixing (κ ≥ 10^−4^ m^2^ s^−1^) have been found over rough topography^8–14^. This study implies that dissipation over rough topography predicted by GHP parameterization might be inaccurate, and the MG parameterization can overcome the defects of GHP parameterization and allow for more accurate intensity and spatial distribution of turbulent diapycnal mixing in the open ocean. One challenge of the application of these fine-scale parameterizations is the necessary local adjustment of *ε*_0_. In spite of this, our result shows that the range of *ε*_0_ for MG parameterization is smaller than that for GHP parameterization (0.75 × 10^−9^ $\le \,{\varepsilon }_{0}^{MG}\,\le$ 4.19 × 10^−9^ W kg^−1^ versus 0.30 × 10^−10^ $\le \,{\varepsilon }_{0}^{GHP}\,\le$ 10.47 × 10^−10^ W kg^−1^). A small range of *ε*_0_ for MG parameterization implies that using the MG parameterization would lead to a smaller distortion in the broad mapping of dissipation rates than using the GHP parameterization if an averaged *ε*_0_ were adopted in the parameterizations. Yet our observation only covers a small region of the Pacific. In order to complete the assessment of fine-scale parameterizations, more extensive simultaneous measurements of fine-structure and microstructure are needed in the near future.

## Methods

### Data

Microstructure data were obtained with an expendable Vertical Microstructure Profiler (VMP-X), a free-falling instrument ballasted to fall at 0.6–0.8 m s^−1^. The instrument was equipped with standard turbulence sensors for measuring turbulent velocity shear (*∂u*/*∂z* and *∂v/∂z*), temperature, and pressure^40^. The maximum measurement depth is 6000 m, which satisfied the maximum depths of the stations (Fig. 1a). Fine-structure temperature and salinity data were collected using a Seabird 9–11 Plus CTD. The CTD data were processed according to standard procedures as recommended by the manufacturer, and bin averaged to 2-m resolution. Buoyancy frequency was computed from finite differencing with the CTD temperature and salinity data. Fine-structure velocity data was measured with two lowered acoustic Doppler current profilers (an upward 300 kHz LADCP and a downward 300 kHz LADCP) mounted on the CTD package. Vertical bin size of LADCPs was 10 m and there were 20 bins for each LADCP. Velocity profiles were processed on a 10-m depth grid with software developed at the Lamont-Doherty Earth Observatory, Columbia University. Baroclinic velocity was computed by removing the depth-mean of each velocity component. Shear was calculated by first-differencing velocity over 10-m intervals.

### Observed dissipation

The observed dissipation rate (*ε*_*OB*_) is calculated by integrating the turbulent velocity shear spectrum *ψ*(*k*)

6 $${\varepsilon }_{OB}=7.5\nu \langle (\frac{{\rm{\partial }}u}{{\rm{\partial }}z}{)}^{2}\rangle =7.5\nu {\int }_{{k}_{1}}^{{k}_{2}}\psi (k)\,dk,$$

where *ν* is the kinematic viscosity and < >denotes the spatial average. The lower integration limit, *k*_1_, is set to 1 cpm, and the upper limit, *k*_2_, is the highest wavenumber not contaminated by vibration noise. Shear spectrum is computed from the shear signal with half-overlapping 6-m depth segments, thus the vertical resolution of *ε*_*OB*_ is 3 m. The noise level^40^ of the VMP-X is 10^−11^ W kg^−1^.

### MG parameterization

MG dissipation rate (*ε*_*MG*_) can be expressed in terms of fine-scale shear and stratification as^18^

7 $${\varepsilon }_{MG}={\varepsilon }_{0}(\frac{N}{{N}_{0}})(\frac{S}{{S}_{0}}),$$

where *S*_0_ = *N*_0_ = 3 cph and *ε*_0_ is an adjustable constant that tunes the parameterized dissipation rate to the observational data. *ε*_*MG*_ are estimated with the 10-m shear and buoyancy frequency; thus *ε*_*MG*_ have a vertical resolution of 10 m. MG parameterization is found to be not sensitive to the shear resolution. It can also reproduce the observed dissipation with low shear resolution, e.g., 20-m shear, 50-m shear (not shown). *ε*_0_ in equation (7) shows variability in different regions^19–21,23^, spanning from 10^−10^ to 10^−8^ W kg^−1^. In order to fit the parameterized dissipations best to the observed dissipations, for each station *ε*_0_ is set to the value that minimizes the sum of squared residuals (a residual being the difference between an observed value and the parameterized value). *ε*_0_ are 0.75 × 10^−9^, 3.12 × 10^−9^, 2.92 × 10^−9^, 3.08 × 10^−9^, 2.01 × 10^−9^, 4.19 × 10^−9^, and 1.89 × 10^−9^ W kg^−1^ for stations A1–A7, respectively, with a mean of 2.57 × 10^−9^ W kg^−1^.

### GHP parameterization

GHP dissipation rate (*ε*_*GHP*_) depends on fine-scale shear and strain variances as^5,6,8^

8 $${\varepsilon }_{GHP}={\varepsilon }_{0}{(\frac{N}{{N}_{0}})}^{2}\frac{{\langle {V}_{z}^{2}\rangle }^{2}}{{}_{GM}\langle {V}_{z}^{2}{\rangle }^{2}}{h}_{1}({R}_{\omega })j(\frac{f}{N}),$$

where *ε*_0_ is the reference dissipation rate, $\langle {V}_{z}^{2}\rangle$ is the observed shear variance, ${}_{GM}\langle {V}_{z}^{2}\rangle$ is the shear variance from the GM model^41^,

$$\begin{array}{c}{h}_{1}({R}_{\omega })=\frac{3({R}_{\omega }+1)}{2\sqrt{2}{R}_{\omega }\sqrt{{R}_{\omega }-1}},\\ j(f/N)=\frac{farccosh(N/f)}{{f}_{30}arccosh({N}_{0}/{f}_{30})},\end{array}$$

${f}_{30}=f({30}^{{\rm{o}}})$, and *N*_0_ = 3 cph. The shear/strain variance ratio ${R}_{\omega }=\langle {V}_{z}^{2}\rangle /({\bar{N}}^{2}\langle {\xi }_{z}^{2}\rangle )$ is a measure of the internal wave field’s aspect ratio and frequency content^6,11^. Strain variance $\langle {\xi }_{z}^{2}\rangle$ is estimated from buoyancy frequency, $\langle {\xi }_{z}^{2}\rangle =\langle {({N}^{2}-{\bar{N}}^{2})}^{2}/{\bar{N}}^{4}\rangle$.

To quantify shear $\langle {V}_{z}^{2}\rangle$ and strain $\langle {\xi }_{z}^{2}\rangle$ variances for GHP parameterization, shear and strain profiles are broken into half-overlapping 300-m-long segments starting from the bottom. Buoyancy-frequency- normalized shear variance $\langle {V}_{z}^{2}\rangle /{\bar{N}}^{2}$ are quantified by integrating the buoyancy-frequency-normalized shear spectra $S[{V}_{z}/\bar{N}]({k}_{z})$ from the lowest resolved wavenumber to a maximum wavenumber that avoids contamination by instrument noise at higher wavenumbers^11^,

$$\frac{\langle {V}_{z}^{2}\rangle }{{\bar{N}}^{2}}={\int }_{{\rm{\min }}\,{k}_{z}}^{{\rm{\max }}\,{k}_{z}}S[{V}_{z}/\bar{N}]({k}_{z})d{k}_{z}.$$

The GM model shear variances used to normalize the observed variances are computed over the same wavenumber band,

$$\frac{{}_{GM}\langle {V}_{z}^{2}\rangle }{{\bar{N}}^{2}}=\frac{3\pi {E}_{0}b{j}_{\ast }}{2}{\int }_{{\rm{\min }}\,{k}_{z}}^{{\rm{\max }}\,{k}_{z}}\frac{{k}_{z}^{2}}{{({k}_{z}+{k}_{{z}^{\ast }})}^{2}}d{k}_{z},$$

where ${k}_{{z}^{\ast }}=\pi {j}_{\ast }N/b{N}_{0}$, ${j}_{\ast }$ = 3, *b* = 1300 m, *N*_0_ = 3 cph, and *E*_0_ = 6.3 × 10^−5^. $\langle {\xi }_{z}^{2}\rangle$ are obtained by integrating the strain spectra,

$$\langle {\xi }_{z}^{2}\rangle ={\int }_{{\rm{\min }}\,{k}_{z}}^{{\rm{\max }}\,{k}_{z}}S[{\xi }_{z}]({k}_{z})d{k}_{z}.$$

**Figure 7:**
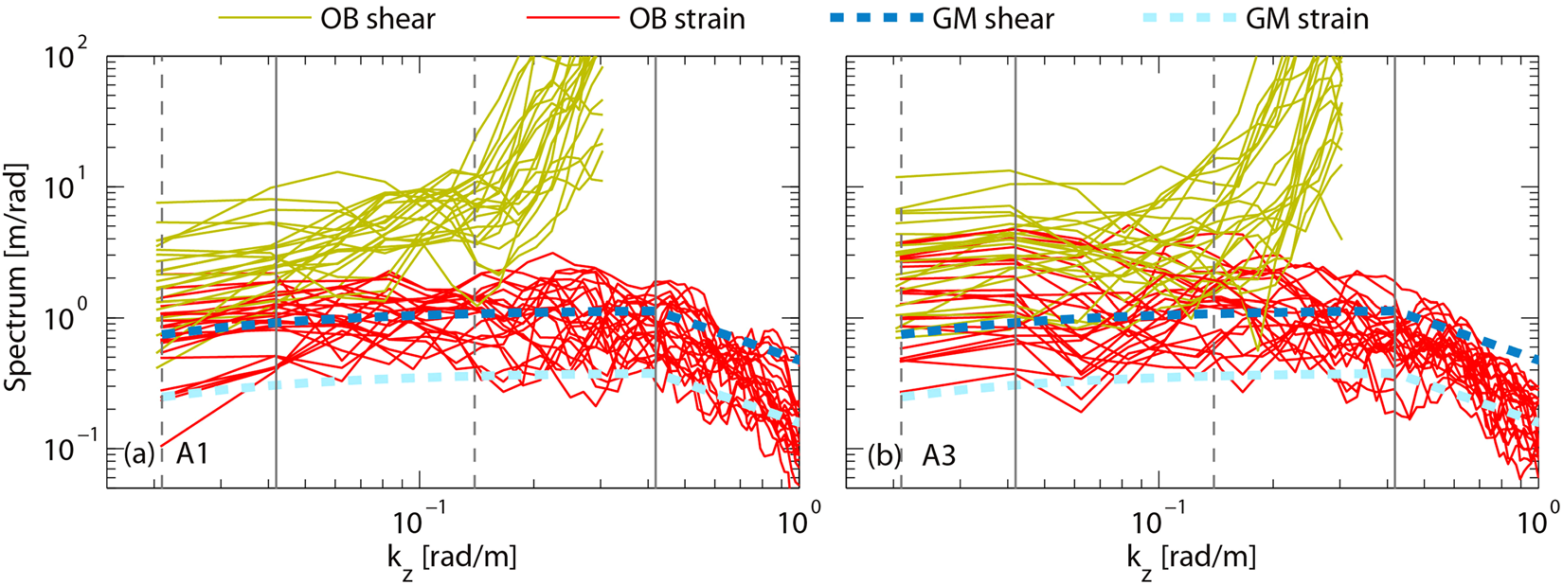
Sample vertical wavenumber spectra for the observed strain (OB strain), observed buoyancy- frequency-normalized shear (OB shear), GM shear, and GM strain from (**a**) station A1 and (**b**) station A3. Vertical dotted (solid) lines demark the integration limits for shear (strain) variance.

Figure 7a,b display samples of vertical wavenumber spectra for $\langle {\xi }_{z}^{2}\rangle$ and $\langle {V}_{z}^{2}\rangle /{\bar{N}}^{2}$, along with the GM spectra. These observed spectra have been corrected with spectral correction functions^11^. Shear spectra become increasingly blue at higher wavenumbers (*k*_*z*_ > 0.140 rad m^−1^) because of instrument noise, so the upper bound for the shear variance integration is set to 0.140 rad m^−1^, corresponding to vertical wavelength *λ*_*z*_ = 45 m. The lower bound for the shear variance integration is the lowest resolved wavenumber (0.021 rad m^−1^), corresponding to vertical wavelength of 300 m. The strain spectra roll off roughly as ${k}_{z}^{-3/2}$ at the high wavenumbers (*k*_*z*_ > 0.419 rad m^−1^). The upper bound for the strain variance integration is set to 0.419 rad m^−1^, corresponding to *λ*_*z*_ = 15 m. The lower bound for the strain variance integration is set to 0.042 rad m^−1^ (corresponding to *λ*_*z*_ = 150 m) because strain variance at the low wavenumbers (*k*_*z*_ < 0.042 rad m^−1^) might be influenced by background stratification. With the obtained $\langle {V}_{z}^{2}\rangle$ and $\langle {\xi }_{z}^{2}\rangle$, *ε*_*GHP*_ are calculated with equation (8), which have a vertical resolution of 150 m. The reference dissipation rate *ε*_0_ in equation (8) shows variability in different studies^8,10–13,16^, spanning from 10^−10^ to 10^−9^ W kg^−1^. In order to fit the parameterized dissipations best to the observed dissipations, for each station *ε*_0_ is set to the value that minimizes the sum of squared residuals. *ε*_0_ are 0.76 × 10^−10^, 3.31 × 10^−10^, 4.68 × 10^−10^, 4.43 × 10^−10^, 4.07 × 10^−10^, 10.47 × 10^−10^, and 0.30 × 10^−10^ W kg^−1^ for stations A1–A7, respectively, with a mean of 4.0 × 10^−10^ W kg^−1^.

### Data availability

The research data can be accessed from the corresponding author Xiao-Dong Shang, whose email is xdshang@scsio.ac.cn.
